# Growth of Nitrogen Incorporated Ultrananocrystalline Diamond Coating on Graphite by Hot Filament Chemical Vapor Deposition

**DOI:** 10.3390/ma15176003

**Published:** 2022-08-31

**Authors:** Daniel Villarreal, Jyoti Sharma, Maria Josefina Arellano-Jimenez, Orlando Auciello, Elida de Obaldía

**Affiliations:** 1 Materials Science and Engineering, University of Texas at Dallas, Richardson, TX 75080, USA; 2 Materials Science and Engineering and Bioengineering, University of Texas at Dallas, Richardson, TX 75080, USA; 3 Facultad de Ciencias y Tecnología, Universidad Tecnológica de Panamá, Panamá City 0819, Panama; 4 Centro de Estudios Multidisciplinarios en Ciencias, Ingeniería y Tecnología-AIP (CEMCIT-AIP), Panamá City 0819, Panama

**Keywords:** hot-filament-chemical-vapor-deposition, natural graphite cooper anodes, nitrogen-incorporated-ultrananocrystalline-diamond-films

## Abstract

This article shows the results of experiments to grow Nitrogen incorporated ultrananocrystalline diamond (N-UNCD) films on commercial natural graphite (NG)/Cu anodes by hot chemical vapor deposition (HFCVD) using a gas mixture of Ar/CH_4_/N_2_/H_2_. The experiments focused on studying the effect of the pressure in the HFCVD chamber, filament-substrate distance, and temperature of the substrate. It was found that a substrate distance of 3.0 cm and a substrate temperature of 575 C were optimal to grow N-UNCD film on the graphite surface as determined by Raman spectroscopy, SEM, and TEM imaging. XPS analysis shows N incorporation through the film. Subsequently, the substrate surface temperature was increased using a heater, while keeping the substrate-filament distance constant at 3.0 cm. In this case, Raman spectra and SEM images of the substrate surface showed a major composition of graphite in the film as the substrate-surface temperature increased. Finally, the process pressure was increased to 10 Torr where it was seen that the growth of N-UNCD film occurred at 2.0 cm at a substrate temperature of 675 C. These results suggest that as the process pressure increases a smaller substrate-filament distance and consequently a higher substrate surface temperature can still enable the N-UNCD film growth by HFCVD. This effect is explained by a mean free path analysis of the main precursors H_2_ and CH_3_ molecules traveling from the filament to the surface of the substrate The potential impact of the process developed to grow electrically conductive N-UNCD films using the relatively low-cost HFCVD process is that this process can be used to grow N-UNCD films on commercial NG/Cu anodes for Li-ion batteries (LIBs), to enable longer stable capacity energy vs. charge/discharge cycles.

## 1. Introduction

A key component of Lithium-Ion batteries (LIBs) is the anode. Various anode materials exhibit appropriate reversible insertion of Li ions [1,2], via intercalation in layered carbon atoms-based structures (e.g., natural graphite), adsorption in hard carbons surface layers, and binding on hydrogen (H) atoms in C-H terminated surface layers [3]. In relation to developing coatings for current LIB’s anodes, a transformational Nitrogen (N) atoms grain-boundary incorporated ultrananocrystalline diamond (N-UNCD) film, produced by microwave plasma chemical vapor deposition (MPCVD) [4], exhibits a high density of grain boundaries with open sp^2^ C-bonds inducing N atoms reaction, resulting in electrons release providing grain boundaries electron conduction enabling uniform electrical conductivity through the N-UNCD films [4,5]. In addition, the N-UNCD films exhibit chemical and electrochemical inertness, high mechanical strength, and superior tribological properties [6]. However, the N-UNCD coating, originally produced by MPCVD, involves a relatively expensive MPCVD system. Therefore, it is critical to develop an alternative N-UNCD film growth process involving a less expensive industrial system, capable of growing N-UNCD films on large areas suitable for growing coating on hundreds of LIB’s anodes at a low cost.

Hot Filament Chemical Vapor Deposition (HFCVD) of N-UNCD films represents a less expensive process in comparison to MPCVD. To develop a process for depositing these films on a substrate by HFCVD, a gas mixture of Ar/CH_4_/N_2_/H_2_ is required and the process pressure, filament-substrate distance, and substrate surface temperature must be tuned at appropriate values for growing N-UNCD films. The adjustment of the mentioned variables represents the kinetical stabilization of N-UNCD versus the graphitic structures which is essential to developing the optimum CVD deposition process, as demonstrated previously in the growth of composite 2D indium oxide films [7].

In previous research, a mixture of gases (H_2_ 4.0 sccm/CH_4_ 1.0 sccm/Ar: 5.0 sccm/N_2_ 6.0 sccm), demonstrated the feasibility for growing N-UNCD films by HFCVD [8,9]. However, the effect of filament-substrate distance, thus travel distance for precursor species described above, the temperature of the substrate surface, and gas pressure to grow N-UNCD films, have not been studied. These parameters correspond to adjusting the kinetics conditions for the growth N-UNCD. Recent articles have theoretically demonstrated that it is possible to tune the appropriate conditions to grow UNCD by varying the kinetic energy of the precursor molecules and atoms. This variation in kinetic energy is achieved by varying the pressure of the process [10]. Additionally, it has been demonstrated experimentally that the travel distance of precursor species affects the prevalence of graphitic phases [10].

In that sense, a previous study on the influence of travel distance of precursor species and surface temperature in the growth of UNCD films by HFCVD using a high CH_4_/H_2_ ratio showed that at smaller substrate-filament distances graphitic phases are favored [9]. On the other hand, a lower CH_4_/H_2_ ratio and low pressures favor the growth of graphite by HFCVD. This happens because hydrogen is responsible for etching the graphitic phases. In gas mixtures with high CH_4_/H_2_ the hydrogen graphite etching rate is poor to overcome the graphite formation rate [10]. Once graphite is formed then it will represent an energy barrier for the diamond phase to nucleate because of the mismatch of diamond and graphite lattice and eventually a film of crystallite graphite will dominate the growth [10]. Nonetheless, as mentioned before, previous research showed that by varying the filament-substrate distance and consequently the temperature of the substrate surface is possible to find the appropriate conditions to favor the growth of UNCD versus graphite films by HFCVD. In this respect, the effect of substrate-filament distance, substrate temperature, and process pressure are analyzed to produce the deposition of N-UNCD films on NG/Cu anodes for LIBs by HFCVD.

## 2. Materials and Experimental Methods

Cooper (Cu) anodes from MTI Corporation (Richmond, CA, USA) were coated on both sides with Natural Graphite (NG) powder. The NG/Cu anodes consisted of a foil of Cu (11 µm thick) covered on both sides with a layer of NG (10 µm thick). One NG layer was exposed to the gas mixture flow in the HFCVD system, during the N-UNCD film growth process, and the other was in contact with the substrate holder (back layer). The NG back layer prevents bending caused by the thermal expansion of the Cu foil during heating before film growth and cooling after film growth.

### 2.1. Seeding of Natural Graphite (NG) Surface with Nanocrystalline Diamond Particles

**Figure 1:**
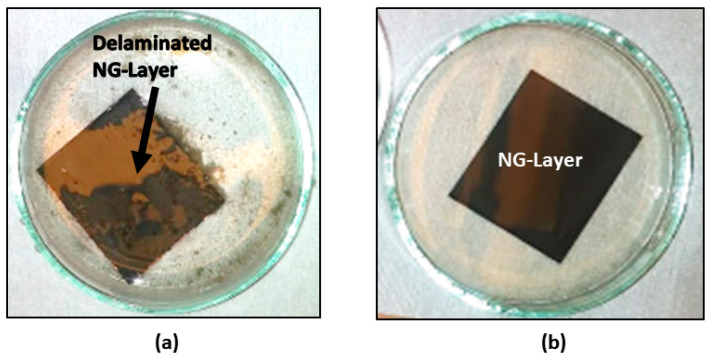
(**a**) NG layer removed from the surface of Cu foil during the seeding process, resulting from ultrasonic agitation with high level fluid; (**b**) NG/Cu substrate with NO NG layer delamination by using solution with fluid surface level just above the substrate surface.

To grow N-UNCD films by HFCVD, commercial UNCD particles (3–5 nm) are inserted on the surface of substrates, in a process named “seeding” [6] to induce the growth of diamond films. Seeding involves immersion of the substrate in a methanol/UNCD particles suspended solution in a “sonicator” system where waves insert the UNCD particles on the substrate surface. A problem was that initial ultrasonic agitation in high volume level solution resulted in delamination of the NG layer from the underlying Cu foil (see Figure 1a). This problem was solved by reducing the solution level with the liquid surface just above the substrate surface, which reduce the strength of liquid waves on the substrate surface, practically eliminating the delamination of the NG layer from the Cu foil (Figure 1b).

### 2.2. HFCVD Controlled Process Parameters for Growing N-UNCD Films

N-UNCD films were grown on NG/Cu substrates with shapes/dimensions of LIBs’ anodes, using the HFCVD process, implemented in a commercial HFCVD system (BlueWave Semiconductor, Baltimore, MD, USA) with the capacity to grow diamond films on up 100 mm diameter substrates. The NG/Cu anodes were heated to achieve an NG surface temperature between 600–675 °C. This was estimated from a temperature calibration curve obtained via measurement with thermocouples on a substrate surface and on the substrate holder surface. The mixture of gases, used to provide the chemical process to grow the N-UNCD films, was: H_2_ (4 sccm), CH_4_ (1 sccm), Ar (5 sccm), N_2_. (6 sccm). The CH_4_, H_2_, and N_2_ molecules flowing into the air evacuated chamber of the HFCVD system, crack upon impact on the hot surface of an array of tungsten (W) filaments (see Figure 2b in Ref. [9]), positioned above the surface of the substrate (distance explored: 1–3 cm), heated to about 2300 °C, via electronic current. C, H, N, and Ar atoms, flowing from the hot filaments to the substrate surface (see Figure 2a in Ref. [9]), interact synergistically, inducing the chemical reactions that grow the N-UNCD films (see Figure 2c in Ref. [9]). The pressure was varied between 10 Torr and 5 Torr to determine optimal gas pressure during film growth conditions. For films grown at constant at 5.0 Torr the filament-substrate distance varied between 1, 2, and 3 cm. All N-UNCD films were grown for two hours to set a uniform film thickness.

Overall, 7 samples were prepared (see Table 1). Samples’ preparation involved tailored substrate surface temperature, filaments-substrate distance, and gas pressure on the growth of N-UNCD films on NG/Cu anodes.

The type of carbon chemical bonding in the films was analyzed via visible Raman spectroscopy using a Thermo DXR Raman spectrometer (Thermo Fisher Scientific, Waltham, MA, USA), with a 532 nm wavelength laser beam. The surface morphology of the samples was characterized using scanning electron microscopy (SEM, ZEISS SUPRA-40, Zeiss Corporation, Oberkochen, Germany). High-Resolution Transmission Electron Microscopy (HRTEM) was performed using an HRTEM (JEOL ARM200F, Akishima, Tokyo, Japan) system, operated at 200 keV. The FFT diffraction from the images was performed with the Digital Micrograph software from the Gatan Microscopy Suite (GMS-3, Pleasanton, CA, USA). XPS analysis was performed using a Versa Probe II (VPII) XPS apparatus from Physical Electronics, Chanhassen, MN, USA, at a base pressure of 4 × 10^−8^ Pa. Data were collected with a pass energy of 1.175 eV.

## 3. Experimental Results

### 3.1. Characterization of Chemical and Surface Morphological Structures of Virgin NG/Cu Foil Anode

**Figure 2:**
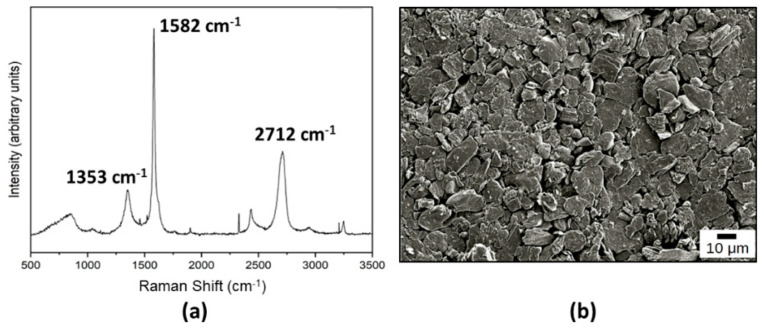
(**a**) Raman spectrum of a virgin NG layer in the fabricated NG/Cu anode; (**b**) SEM image of the surface of the NG layer characterized by the Raman analysis shown in (**a**).

**Figure 3:**
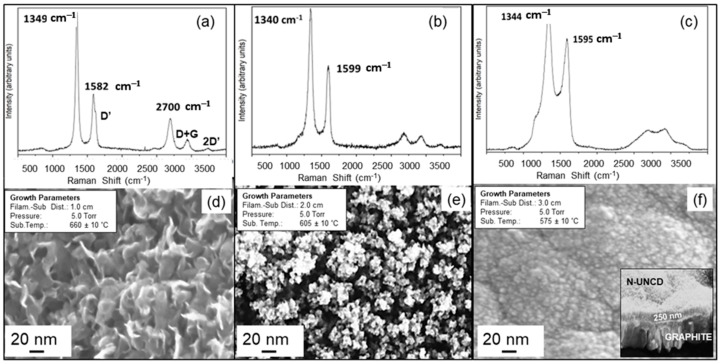
Raman spectrum (visible 532 nm laser beam wavelength) from sample 1 (**a**), sample 2 (**b**), sample 3 (**c**), respectively; SEM images from the surface of sample 1 (**d**), sample 2 (**e**), sample 3 (**f**). (**f**) shows surface morphology characteristic of N-UNCD very thin film (−250 nm, shown in cross-section SEM bottom right image insert in (**f**)). The N-UNCD film with Raman and SEM images shown in (**c**,**f**), respectively, was grown at 5 Torr, filaments-substrate distance of 3 cm, and substrate temperature of 575 °C.

For reference, a virgin NG/Cu foil anode was analyzed by Raman Spectroscopy to determine the chemical nature of the NG layer (Figure 2a), and by scanning electron microscopy (SEM), to determine the morphology of the virgin NG surface (Figure 2b), both of which can have a relevant effect on Li ions interaction in the anode of a LIB. The Raman spectrum shows the characteristic structure of graphite/defected graphene multilayers [11,12,13], with the peak at 2712 cm^−1^ lower than the 1582 cm^−1^ peak. On the other hand, the SEM image (Figure 3b) shows the well-known turbostratic disordered and unorganized or buckled layers of graphite [11].

### 3.2. Characterization of Chemical and Surface Morphological Structures of N-UNCD Film Grown on Seeded NG Layer on Cu Foil Anode

The proper conditions to grow N-UNCD films on NG/Cu anodes by HFCVD, depends strongly on the NG surface temperature correlated with the combined substrate heating plus filament radiation, the latter depending substantially on the filament-substrate distance. In addition, the CH_4_/H_2_ gases ratio has a key role since this ratio controls the growth of graphitic and diamond phases when growing UNCD or N-UNCD films [6,14]. Raman analysis was used to determine the chemical bonds of C atoms in the N-UNCD films. The growth of the N-UNCD films described in this section was done using the same H_2_ (4 sccm), CH_4_ (1 sccm), Ar (5 sccm), and N_2_ (6 sccm) gas mixture, changing the filament-substrate distance, NG surface temperature, and growth gas pressure as indicated in Table 1 for samples 2, 3, and 4.

**Figure 4:**
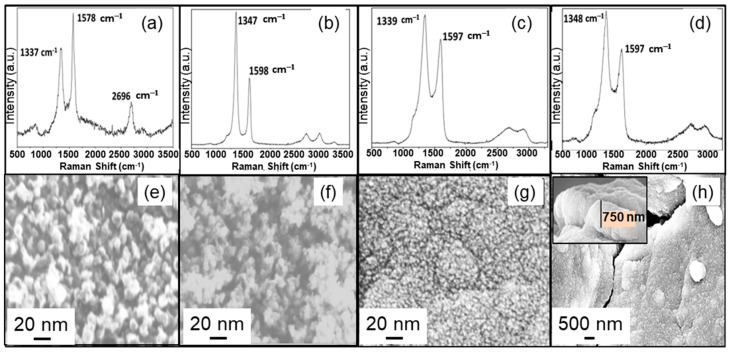
Raman spectrum (visible 532 nm laser beam wavelength) from N-UNCD films for sample 4 (**a**), sample 5 (**b**), sample 6 (**c**), and sample 7 (**d**). SEM plan-view (**e**–**h**) corresponds to films of samples 4, 5, 6, and 7, respectively. N-UNCD films for samples 4 and 5 were grown at 5 Torr and 625 °C and 675 °C substrate temperature, respectively at 3 cm from the filaments. N-UNCD related to samples 6 and 7 were grown at 10 Torr, 650 °C, at 2 cm from filaments. N-UNCD film for sample 7 was grown for 4 h.

***Complementary Raman-Chemical and SEM-Morphological Analysis of N-UNCD Films:*** The Raman Spectrum of sample 2 (Figure 3a) exhibits three prominent peaks at 1349 cm^−1^ (D band), 1582 cm^−1^ (G band), and 2700 cm^−1^ (2D band). Correlated with the Raman spectrum in Figure 3a, an SEM plan-view image (Figure 3d) shows a structure characterized by carbon-based standing platelets, which is correlated with structures observed before for UNCD films grown by HFCVD with filament-substrate distance in the range 1–1.5 cm [6]. These platelet structures are correlated to graphene nanowalls (GNW) which correspond to a few layers of corrugated graphene standing vertically on the substrate, which is supported by the Raman Spectrum in Figure 3a, where the D’ prime band near to the G peak at 1582 cm^−1^ and the presence of the D + G and 2 D’ peak to the right of the 2D band (2700 cm^−1^) [15]. The Raman Spectrum from sample 3, in Figure 3b, exhibits peaks at 1340 cm^−1^ (D band) and 1599 cm^−1^ (G band), with the bottom of the peaks higher than in Figure 3a, while the peak at 2700 cm^−1^ decreased substantially. Based on prior research [4], the change in the Raman peak intensity observed in Figure 3b correlates with the initial nucleation of low-density N-UNCD layers mixed with the graphite base. An SEM image of the N-UNCD film’s surface (Figure 3e), correlated to the Raman image in Figure 3b, shows a mixture of granulated carbon structure (black areas) with initial N-UNCD grain nucleation (light areas). Figure 3c (Raman spectrum), and corresponding Figure 3f (SEM Image of N-UNCD film’s surface) corresponds to the N-UNCD film of sample 4, grown by HFCVD with filament-substrate distance at 3 cm and NG surface temperature at 575 °C. The Raman spectrum reveals peaks at 1345 cm^−1^, which encapsulates the 1332 cm^−1^ peak correlated with sp^3^ C atoms bonds characteristic of diamond in the grains, and sp^2^ bonds of C atoms in the grain boundaries, expected for N-UNCD films, as shown in those grown by MPCVD [16]. The Raman spectrum shown in Figure 3c and Figure 4c,d are considered N-UNCD thin films, since they show the TPA peaks at 1150 ane1480 cm-1 characteristic of UNCD films [17], although the D and G peaks are a bit sharper and higher than the peaks observed in thick N-UNCD films produced by MPCVD [5,6] or thicker N-UNCD films grown recently by HFCVD [8,18] on Si substrate. This may be due to the laser beam reaching the underlying graphite layer used as substrate. For Figure 3c and Figure 4a,b the growth of N-UNCD is not a continuous layer, as can be appreciated in Figure 3e and Figure 4e,f, which allows a larger contribution of the graphite substrate to be exposed and show up in the Raman spectrum as a sharp peak for the D and G bands. The presence of a small TPA signature shows some N-UNCD growth. As the pressure and gas flows were kept constant throughout the process, the variation in the chemistry of the deposited film is related to the influence of the traveling distance of the species and the temperature of the substrate’s surface.

To analyze the influence of NG surface temperature on the growth of the N-UNCD film on NG/Cu anode, N-UNCD films were grown with the optimized H_2_ (4 sccm)/CH_4_ (1 sccm)/Ar (5 sccm)/N_2_ (6 sccm) gas mixture flow, previously demonstrated by our group, with a filament-substrate distance of 3 cm and surface temperature of 625 °C and 675 °C (samples 4 and 5 in Table 1). The Raman Spectra from samples 4 and 5 (Figure 4a,b), respectively, show peaks characteristic of graphitic structures, as observed for the films of samples 1 and 2, grown at 1 cm and 2 cm filament-substrate distance and relatively high substrate temperature (650 °C and 605 °C, respectively) (Figure 3a,b). The Raman Spectrum for samples 4 and 5, showing the graphitic structure, suggests that high substrate temperature may induce a similar graphitic structure as produced at a short filament-substrate distance, which can induce high substrate surface temperature due to m more efficient radiation on the substrate surface from the filaments.

Another set of experiments was performed to explore the effect of pressure on the growth of N-UNCD films. Sample 6 relates to an N-UNCD film grown on an NG/Cu anode at 10 Torr pressure, a filament-substrate distance of 2 cm, and an NG surface temperature of 650 °C. The Raman spectrum (Figure 4c) and SEM Surface image (Figure 4g) of Sample 6, show the Raman spectrum and surface morphological structure characteristic of N-UNCD, as for sample 3 for which the N-UNCD film was grown at different deposition parameters. These results suggest that there is compensation between the temperature and traveling distances to enable the growth of N-UNCD films, via HFCVD, under different pressures.

**Figure 5:**
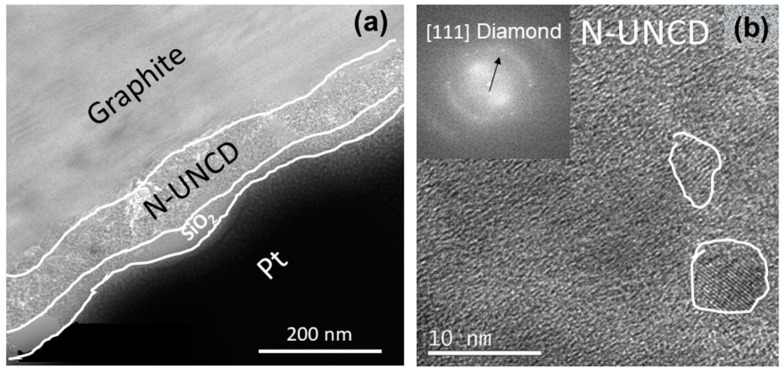
High-resolution TEM of N-UNCD grown on graphite. (**a**) Cross section TEM (**b**) high-resolution image with FFT diffraction grating showing the plane distance corresponding to [111] diamond plane.

***Complementary High-Resolution TEM:***Figure 5a shows the HRTEM image of a cross-section of a graphite/N-UNCD sample. To prepare the sample for the TEM, a thin layer of SiO2 was deposited, followed by a thicker layer of platinum. There is a sharp interface between the graphite and the NUNCD. The high-resolution image in Figure 5b shows the presence of diamond grains, further corroborated with the FTT analysis of the area which shows the miller plane distance corresponding to [111] diamond.

**Figure 6:**
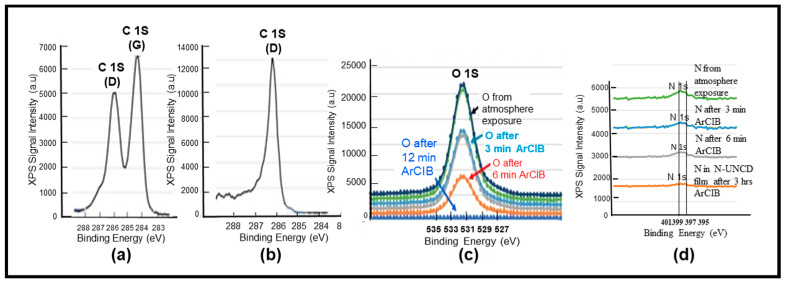
XPS spectra of key atoms in the N-UNCD film grown on NG/Cu anode: (**a**) C 1s peaks (G-related to the sp^2^ C bonds characteristic of graphite [19]) corresponding to adventitious C atoms from the atmosphere adsorbed on the N-UNCD film surface/(D- related to the sp^3^ C bonds characteristic of diamond [19]); (**b**) C 1s peak after 12 min ArCIB bombardment, (**c**) O 1s peak, corresponding to O atoms adsorbed from the atmosphere, which disappear after 12 min ArCIB bombardment; (**d**) N 1s peak (double structured), revealing N atoms adsorbed from the atmosphere, which are practically eliminated after 6 min ArCIB bombardment, with the remaining small N 1s intensity peak, after 3 h. ArCIB bombardment related to N atoms inserted in the N-UNCD film during growth.

***Complementary X-ray Photoelectron Spectroscopy (XPS)-Chemical Analysis of N-UNCD Films:*** XPS analysis of N-UNCD film grown on NG/Cu anodes was performed to determine the chemical bonds of the C and N atoms at the nanoscale from the film surface. To obtain accurate XPS analysis of samples exposed to the atmosphere before insertion in the XPS system, it is critical to clean the sample’s surface from species adsorbed from the atmosphere. The surface cleaning was done via bombardment with an Argonne (Ar) atoms-based Cluster Ion Beam (ArCIB), integrated into the XPS system, formed by 2500 Ar atoms in the CIB^+^ with 20 keV energy, resulting in individual Ar atoms with 8 eV energy upon cracking of the CIB when impacting the sample’s surface, inducing ejection of impurity atoms from the surface, without damaging the surface and avoiding Ar incorporation as it occurs when bombarding with an energetic (2–5 keV) single Ar^+^ ion beam [19]. The N-UNCD surface of the coating on the NG/Cu anode was bombarded for 3, 6, 12 and 180 min. Figure 6a shows the presence of two C 1s XPS peaks before ArCIB cleaning. The C 1s (G) peak at −284 eV is correlated with adventitious C atoms adsorbed from the atmosphere, which exhibit the characteristic sp^2^ C atoms bonds of graphite. The C 1s (D) peak at −286 eV correlates with the C atoms with sp^3^ bonds characteristic of diamond [19]. The C 1s (G) peak disappeared after only 1 min bombardment, revealing the cleaning of the N-UNCD film surface from adventitious C and revealing the C 1s (D) peak of diamond (Figure 6b,c) shows the O 1s peak, which disappeared after 12 min ACIB bombardment. Figure 6d shows the key N 1s double structured peak evolution vs. ArCIB bombardment. The N 1s XPS signal (double structured peak) reduces substantially after 6 min bombardment, due to the elimination of atmospheric surface adsorbed N atoms. However, the signal correlated with N atoms inserted in the grown N-UNCD film is present after ~ 3 h. bombardment. The signal for N 1s in the XPS is very weak, but its presence is evidence of N incorporation in N-UNCD.

## 4. Discussion

As was initially expected, the seeding process carried out, before N-UNCD film growth, is necessary for the nucleation of N-UNCD films on NG/Cu anodes, since HFCVD requires to have a high density of nucleation sites for the growth of UNCD films [17,20]. The Raman spectrum of samples without seeding (not shown) showed no difference compared to that of the virgin NG/Cu anode (Figure 2a).

Related to the growth of UNCD films, on NG/Cu Anodes, via HFCVD, a previous study demonstrated the influence of the traveling distance of the C and CHx species from the filaments to the substrates’ surface and the substrates’ temperature during low-temperature growth of UNCD films by HFCVD [9]. In addition, other studies demonstrated that the growth of UNCD or Graphite depends on the CH_4_/H_2_ ratio [10], where a small ratio will benefit the diamond phase because the H atoms will be abundant to etch the graphite phase, which etches preferentially with respect to the diamond phase, due to open C atoms bond on the graphite phase, as opposed to the diamond phase where all four C atoms bonds are linked to other C atoms. On the other hand, a high CH_4_/H_2_ ratio will make the graphite etching rate decrease because there is less atomic H.

In the research described in this article, the ratio of H_2_/CH_4_ is 4 (as calculated from the gas flows) which represents a large ratio. The Raman spectrum of samples 2 and 3 (see Figure 3a,b) have prominent G peaks indicating the presence of graphite on the samples. On the other hand, sample 4 (see Figure 3c,f), grown at a 3 cm filament-substrate distance, exhibits the Raman spectrum characteristic of the N-UNCD film. These can be interpreted as a result that a large H_2_/CH_4_ ratio would induce larger C atoms than H atoms concentration, resulting in substantially reduced etching of the co-deposited graphite phase. As the filament-substrate distance increases, the availability of the precursor atoms and molecules (H, C, CH_x_, N) with sufficient energy, to reach and react on the substrate surface, decreases. The energy is reduced because the molecules must travel a longer distance between filaments and the substrate surface, which implies more collisions with other molecules in the gas phase in the growth chamber. Atomic H is less affected since it is the atom with the smallest mass and dimension, thus losing less energy while moving towards the substrate surface, resulting in more atomic H for reaction with C atoms with open sp^2^ bonds characteristic of graphite phase that may be trying to grow. Therefore, there is a substantial chemical etching of the graphite phase, resulting in favor of diamond phase growth. Calculations were made comparing the mean free path of atomic H versus the mean free path of CH_3,_ which are considered the major carbon precursor for UNCD film growth [9,10,21,22].

The mean free path (*λ*) is inversely proportioned to the squared effective diameter (d) of a particle, i.e.,

(1) $$\lambda =\frac{kT}{\sqrt{2}\pi d^{2}P}$$

where *P* is the gas pressure, *d* is the effective diameter of species (e.g., H and CH_3_) in the gas phase, *k* is the Boltzmann constant, and *T* is the absolute temperature in the gas mixture, which in prior research [9,22] was interpreted as being close to the filament’s temperature. Therefore, if Equation (1) is used to estimate the mean free path of atomic H and CH_3_ molecules, for a certain temperature and pressure, and then the ratio $\lambda_{CH_{3}}/\lambda_{H}$ is established mathematically, the mean free path ratio of two components is equal to the squared inverse ratio of the effective diameter of H and CH_3_, as shown in Equation (2) below,

(2) $$\lambda_{{\mathrm{CH}}_{3}}={\left(\frac{d_{H}}{d_{{\mathrm{CH}}_{3}}}\right)}^{2}\lambda_{H}$$

where, $\lambda_{{\mathrm{CH}}_{3}}$ is the CH_3_ mean free path, $d_{{\mathrm{CH}}_{3}}$ is the effective diameter of CH_3_, $d_{H}$ is the atomic H effective diameter and $\lambda_{H}$ is the atomic H mean free path. Using the Van der Waals Radius for atomic H and CH_3_, a rough comparison of the mean free paths of H and CH_3_ can be obtained Using Van der Waals values from the literature [23,24] (Table 2), the relation between $\lambda_{{\mathrm{CH}}_{3}}$ and $\lambda_{H}\;$ can be obtained by Equation (3) below,

(3) $$\lambda_{CH_{3}}=0.36\lambda_{H}$$

The result obtained using Equation (3) highlights the imperative difference in the mean free path for H to CH_3,_ which supports the hypothesis that the CH_3_ molecules face a considerable reduction in the mean free path compared to that of atomic H. These results immediately point out that the CH_3_ molecules face more collisions before arriving at the substrate surface. The calculation of the ratio of mean free paths of CH_3_ and atomic H may enable the determination of the filaments-substrate distance to tune the energetic conditions for an appropriate graphite etching rate to enable a predominant growth of the diamond phase.

That hypothesis and calculation described above may help to explain the results obtained for the growth of N-UNCD films via changes in the filaments-substrate distance. On the other hand, the substrate temperature plays an important role in the predominance of the graphite phase over the diamond phase. This is seen in the graphitic structures on samples 4 and 5 (Figure 4a,d and Figure 4b,f, respectively). For samples 4 and 5, the films were grown at the same filament-substrate distance (3 cm), but at higher substrate temperatures (625 °C and 675 °C, respectively), with respect to films grown in sample 3 (N-UNCD/575 °C)/Figure 3c,f provided by the heater. This result may be explained in terms of the energy of molecules (mainly CH_3_, as calculated) and atoms (H, C, N) arriving at the substrate surface. The higher substrate temperatures imply that the activation energy for the reaction is lowered [25], which causes CH_3_ molecules with lower energy arriving at the substrate surface to readily react. This would cause the rate of etching of the graphite phase to be smaller than the one at a lower temperature for the fixed filament-substrate distance of 3 cm, used for the film growth.

The observations regarding the substrate temperature may provide the means to roughly establish a range of thermal stability for N-UNCD film deposition. This range is expected to be narrow since the variation of the substrate temperature from 575 °C to 625 °C (−50 °C) (see Table 1) at 3.0 cm originated the change of N-UNCD to a dominant graphite presence in the film. This suggests that the range of temperature for what the N-UNCD film at a given pressure and distance can be deposited is under 50 °C.

Higher pressure implies a higher frequency of collisions of particles in a gas phase, which results in the reduction of the mean free path. Equation (3) shows that a reduction of the mean free path will always cause a higher impact on the bigger molecule (CH_3_) as compared to atomic *H*. So, in that sense, the appropriate conditions for growing N-UNCD films can be reached using a shorter filament-substrate distance as the pressure increases, due to decrease in energy and mean-free path ($\lambda_{{\mathrm{CH}}_{3}}$) of the CH_3_ molecules, which as a function of pressure *P*, can be estimated by using Equation (1). To determine the mean free path ($\lambda_{{\mathrm{CH}}_{3}}$) of CH_3_ molecules at different pressures, it is relevant to calculate first the ratio of the mean free path to the ratio of pressures for atomic *H*, as shown in Equation (4) below,

(4) $$\lambda_{H}^{2}=\frac{P_{1}}{P_{2}}\lambda_{H}^{1}$$

where, $\lambda_{H}^{2}$ and $\lambda_{H}^{1}$ are the mean free path of hydrogen at pressures *P*_2_ and *P*_1_. In the case of changing the pressure, during film growth, from 5 to 10 Torr, as done for the research described in this article, the pressure ratio (5/10) will be 0.5. Replacing the values for *P*_1_ = 5 and *P*_2_ = 10 into Equation (4) generates Equation (5),

(5) $$\lambda_{H}^{2}=0.5\lambda_{H}^{1}$$

Then, it is necessary to calculate the mean free path ratio between CH_3_ at pressure *P*_2_ and *H* at pressure *P*_1_ to estimate the influence of the pressure in the mean free paths of CH_3_ at pressure *P*_2_ and *H*, at pressure *P*_1_, as shown in Equation (6) below,

(6) $$\lambda_{{\mathrm{CH}}_{3}}^{2}=0.5*0.36\lambda_{H}^{1}=0.18\lambda_{H}^{1}\;\;$$

Based on Equation (6), it is seen that the mean free path of CH_3_ at 10 Torr is 0.18 times shorter than that of H atoms at 5 Torr. This pressure effect indicates that at a process pressure of 10 Torr, the N-UNCD film grown at a shorter substrate-filament distance (2 cm), which induce stronger filament radiation effect on the substrate surface, increasing the substrate temperature to 675 °C. In this case, the number of H atoms with enough energy for reaction on the substrate surface is high enough to induce etching of the graphite phase, thus promoting the growth of the pure N-UNCD film.

Another critical parameter for the growth of N-UNCD on graphite is the role played by the N_2_ gas flow. XPS results show the incorporation of Nitrogen throughout the N-UNCD film. For the incorporation of N in the films, it requires a constant supply of N radical at the surface of the substrate. This limits the energy space, which must be supplied by the Hot Filament. Nitrogen can also compete with growth sites, making the growth of N-UNCD less efficient than for UNCD [8].

Regarding information on sp2 C-bonds in grain boundaries available for N atoms chemical bonding, the reader is encouraged to read the article recently published by our group, which provides valuable quantitative information on bonds in grain boundaries of UNCD films [17].

## 5. Conclusions

The growth of N-UNCD films on NG/Cu anodes by hot filament chemical vapor deposition (HFCVD) represents an alternative process to the traditional growth of N-UNCD films by the MPCVD process. In the research described in this article, the effect of seeding of diamond nanoparticles, the effect of gas flows in the chamber, the pressure of the process, substrate-filament distance, and the temperature of the substrate’s surface were investigated. The seeding is needed to have enough nucleation sites for the N-UNCD film nucleation and growth. The graphite surface does not represent a surface where N-UNCD nuclei can grow at an appropriate rate. That is believed to happen because of the graphite and diamond lattice size mismatch. In this research, the gas flow was kept constant where the ratio of H_2_/CH_4_ was 4. Based on previous studies [25], a high ratio such as the one used in this study favors the predominant growth of the co-deposited graphite phase. As shown in the research described here, tunning of the appropriate conditions for the growth of N-UNCD films is possible by varying the pressure and substrate-filament distance. It was found that at higher process pressures (10 Torr) the needed substrate-filament distance can be shorter compared to that at lower pressure (5 Torr). It was also demonstrated that the substrate temperature plays an important role since when the appropriate distance is established, the increase in temperature can cause the predominance of the growth of the graphite phase. This is believed to happen because higher surface substrate temperature reduces the activation energy for reactions which causes more CH_x_ precursors to readily react, which may imply that the etching rate of the graphite phase decreases. In general, this research demonstrated that tunning of proper film growth conditions may provide the optimized process to grow N-UNCD films on NG/Cu anodes without graphite impurity phase, using an H_2_/CH_4_ gas ratio of 4. Future studies underway are focused on exploring the conditions for growing optimized N-UNCD films on other substrates for Li-ion batteries anodes.

## Figures and Tables

**Table 1:** Number of NG/Cu anodes (Samples) prepared, substrates’ surface temperature, filaments-substrates’ distance, chamber pressure and growth time.

Sample	Substrate Surface Temperature ± 10 °C	Filament Substrate Distance (cm)	Pressure (Torr)	Growth Time (h)
**1**	660	1.0	10	2.0
**2**	605	2.0	5	2.0
**3**	575	3.0	5	2.0
**4**	625	3.0	5	2.0
**5**	675	3.0	5	2.0
**6**	650	2.0	10	2.0
**7**	650	2.0	10	4.0

**Table 2:** Van der Waals radius of atomic H and CH_3_ [23,24].

Molecule/Atom	Van Der Waals Radius (Å)
**H**	1.2
**CH_3_**	2.0
